# The Role of Diffusion-Weighted Imaging (DWI) in Locoregional Therapy Outcome Prediction and Response Assessment for Hepatocellular Carcinoma (HCC): The New Era of Functional Imaging Biomarkers

**DOI:** 10.3390/diagnostics5040546

**Published:** 2015-11-30

**Authors:** Johannes M. Ludwig, Juan C. Camacho, Nima Kokabi, Minzhi Xing, Hyun S. Kim

**Affiliations:** 1 Division of Interventional Radiology, Department of Radiology and Biomedical Imaging, Yale University, New Haven, CT 06510, USA; E-Mails: johannes.ludwig@yale.edu (J.M.L.); minzhi.xing@yale.edu (M.X.); 2 Division of Interventional Radiology and Image-guided Medicine, Department of Radiology and Imaging Sciences, Emory University School of Medicine, Atlanta, GA 30322, USA; E-Mails: juan.camacho@emory.edu (J.C.C.); nima.kokabi@emory.edu (N.K.); 3 Yale Cancer Center, Yale School of Medicine, New Haven, CT 06519, USA

**Keywords:** diffusion-weighted MRI (magnetic resonance imaging), hepatocellular carcinoma, locoregional therapies, imaging biomarker, therapy outcome

## Abstract

Reliable response criteria are critical for the evaluation of therapeutic response in hepatocellular carcinoma (HCC). Current response assessment is mainly based on: (1) changes in size, which is at times unreliable and lag behind the result of therapy; and (2) contrast enhancement, which can be difficult to quantify in the presence of benign post-procedural changes and in tumors presenting with a heterogeneous pattern of enhancement. Given these challenges, functional magnetic resonance imaging (MRI) techniques, such as diffusion-weighted imaging (DWI) have been recently investigated, aiding specificity to locoregional therapy response assessment and outcome prediction. Briefly, DWI quantifies diffusion of water occurring naturally at a cellular level (Brownian movement), which is restricted in multiple neoplasms because of high cellularity. Disruption of cellular integrity secondary to therapy results in increased water diffusion across the injured membranes. This review will provide an overview of the current literature on DWI therapy response assessment and outcome prediction in HCC following treatment with locoregional therapies.

## 1. Introduction

Hepatocellular carcinoma (HCC) is the 3rd deadliest cancer worldwide [1] with a 3-4-fold incidence increase within the past decades in the United States [2]. The increasing incidence of HCC in Western countries is associated with the high prevalence of Hepatitis C, as well as other causes of chronic liver diseases like the nonalcoholic steatohepatitis [3,4]. Tumor excision and liver transplantation are the preferred treatments for HCC while liver transplantation also allows for management of underlying chronic liver disease. When surgical strategies are not possible, small lesions can potentially be treated curatively with ablative modalities. However, most patients are diagnosed in intermediate or advanced stages and curative modalities are of limited utility. Therefore, intra-arterial locoregional therapies, particularly transarterial chemoembolization (TACE), drug-eluting bead transarterial chemoembolization (DEB-TACE), and Yttrium-90 radioembolization (Y90), play a substantial role in downstaging the disease prior to liver transplantation or as palliative modalities. Furthermore, the systemic administration of the multi-kinase inhibitor Sorafenib represents an additional treatment option when locoregional modalities are not reasonable to pursue or failed [4,5].

Tumor response evaluation after locoregional therapies is critical for patient management, and cross-sectional imaging (CT (computed tomography) or MRI (magnetic resonance imaging)) plays a substantial role in this. Traditional anatomical response criteria (World Health Organization (WHO) criteria and Response Evaluation Criteria in Solid Tumors (RECIST)) are solely based on tumor size changes and have been primarily developed for response evaluation of systemically given cytotoxic agents [6]. In the setting of locoregional therapies for HCC however, the simple evaluation of morphological alterations may not be ideal since relevant size changes for response assessment usually take time to occur [7,8,9,10,11]. Moreover, after local tumor ablation, the measured lesion size can even be increased for a prolonged time period due to effects of ablation on the tumor and the adjacent liver parenchyma [12,13].

Therefore, the modified RECIST (mRECIST) criteria and European Association for Study of the Liver (EASL) criteria were developed with the aim of achieving reliable and early response assessment not solely based on tumor size changes. These criteria determine changes in tumor viability by detection of perfusion changes, using contrast enhanced cross sectional imaging [6,8]. However, since post-therapy contrast enhancement changes are not unique features of tumorous lesions and may also occur in benign post-treatment changes such as adjacent tissue inflammation and granulation [14,15], the utility of these response criteria may be limited. Furthermore, in cases of infiltrative lesions or heterogeneous necrosis, anatomical response criteria may be difficult or even impossible to apply appropriately [16,17,18].

To overcome these shortcomings, technological advancements in functional MR imaging, such as diffusion-weighted imaging (DWI), are increasingly being used and evaluated for both post-treatment response assessment and outcome prediction prior to therapy. The purpose of this review is to:

- Provide an overview of the technical aspects of DWI in the liver;
- Review the current literature investigating the role of DWI for response assessment and outcome prediction in HCC following treatment with locoregional therapies; and
- Discuss potential limitations of DWI imaging.

## 2. DWI (Diffusion-Weighted Imaging) Principles

The concept of DWI was first described in 1965 based on a conventional T2-weighted MR sequence [17]. The first brain diffusion-weighted imaging was reported in 1986 and became commonly used in stroke detection in the early 1990s. To date, DWI has been expanded to many applications outside neuroradiology, including DWI of the liver [19,20]. The technical principle of DWI is based on the motion of water molecules within a measured voxel, also known as Brownian movement. In a homogenous liquid, the movement of water molecules is considered “unrestricted”. Within biological tissues, however, microstructural barriers such as cell membranes, intracellular organelles, macromolecules, and other tissue compartments impede water movement (restriction) [21,22,23,24]. Therefore, tissues with higher cellularity, increased nuclear to cytoplasmic ratio, and the presence of intact cell membranes have a greater diffusion restriction (*i.e.*, neoplasms). Any disruption of cellular integrity (e.g., locoregional therapies) will result in increased water diffusion with a corresponding increase of the Apparent Diffusion Coefficient (ADC) value, a quantitative DWI measurement method [21,23].

From a technical standpoint, DWI is a spin-echo T2-weigthed sequence that has an initial 90° RF (radiofrequency) pulse followed by a 180° RF pulse, with the T2 decay related to transverse relaxation. Measuring water diffusion is possible because of the application of a dephasing gradient (diffusion sensitizing gradient). A symmetric rephasing gradient is then applied after the 180° RF pulse. The relocation of water molecules between dephasing and rephasing results in a reduction of signal intensity on DWI [21,23]. The term *b*-value (s/mm^2^) is referred to the strength of diffusion sensitizing gradient and is proportional to the duration and amplitude of dephasing and rephasing gradient [25]. A *b*-value of 0 s/mm^2^ equals a regular T2-weigthed image (no sensitizing gradient applied), thus, the presence of water molecules will be detected. A small *b*-value (<100 s/mm^2^) will result in a signal loss of the water molecules that move faster as seen in intravascular blood. Tissue with high cellularity, on the other hand, will have zero to minimal signal loss when higher *b*-values are applied (>500 s/mm^2^) [21,23,26]. The qualitative assessment of relative signal loss at different *b*-values can be used to detect and characterize lesions and to assess treatment related changes; quantitative DWI data allows for calculation of values based on the aforementioned concepts. One of the most commonly used quantitative diffusion parameter is the Apparent Diffusion Coefficient (ADC) (mm^2^/s), which is represented by the gradient (slope) of the line obtained by plotting the logarithmic signal decay (*x*-axis) against each *b*-value used for measuring (*y*-axis). At least two *b*-value measurements are needed for ADC value determination [23].

## 3. The Value of Diffusion-Weighted Imaging for Locoregional Therapies

### 3.1. Response Assessment of DWI with Imaging Correlation

#### 3.1.1. Transarterial Chemoembolization (TACE)

**Figure 1:**
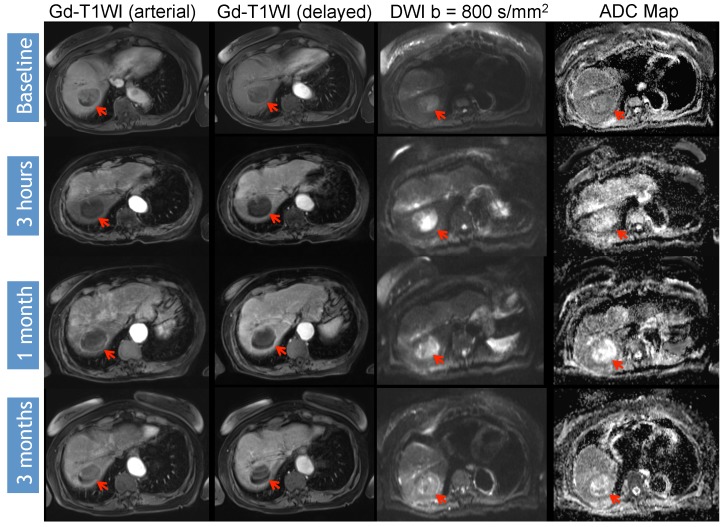
DWI (diffusion-weighted imaging) Response Assessment after DEB-TACE (drug-eluting bead transarterial chemoembolization). 65-year-old man presented with a segment VIII HCC (hepatocellular carcinoma) (arrow) without macrovascular invasion or extrahepatic disease. Note that there is no significant change in the post-treatment arterial Gadolinium (Gd)-enhancement pattern; although there is mild increase in the delayed Gd-enhancement over time. However, when analyzing DWI images, ADC (apparent diffusion coefficient) values increased progressively for ≥20% (baseline ADC 0.808 × 10^−3^ mm^2^/s, post-3 h ADC 1.60 × 10^−3^ mm^2^/s, post-one month ADC 2.30 × 10^−3^ mm^2^/s and post-three month ADC 2.80 × 10^−3^ mm^2^/s), and visually, the central area of restriction is no longer noted. The findings represent a subjective and objective measurement of response, in this case representing complete response (CR) to therapy.

**Figure 2:**
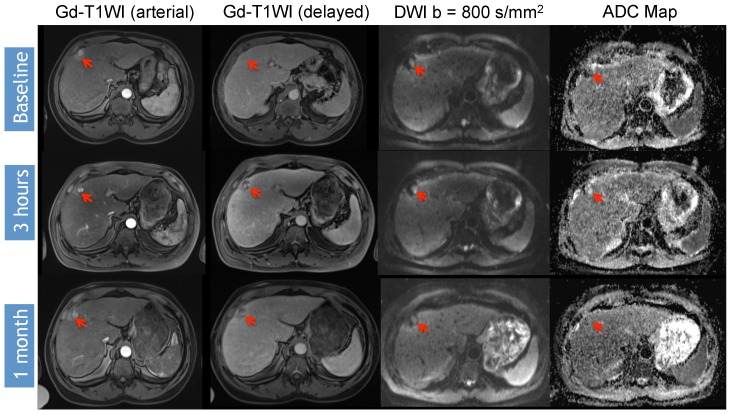
DWI Response Assessment after DEB-TACE treatment. 69-year-old man presented with a segment V HCC (arrow) without macrovascular invasion or extrahepatic disease. Note that there is decreased peripheral enhancement at 3 h that becomes more evident at one month. When analyzing DWI images, ADC values increased ≥20% in the treated region (baseline ADC 0.704 × 10^−3^ mm^2^/s, post-3 h ADC 1.30 × 10^−3^ mm^2^/s); however, a small nodule of restricted diffusion persists at one month (ADC 0.940 × 10^−3^ mm^2^/s). The findings represent a subjective and objective measurement of response, in this case representing partial response (PR) to therapy.

**Figure 3:**
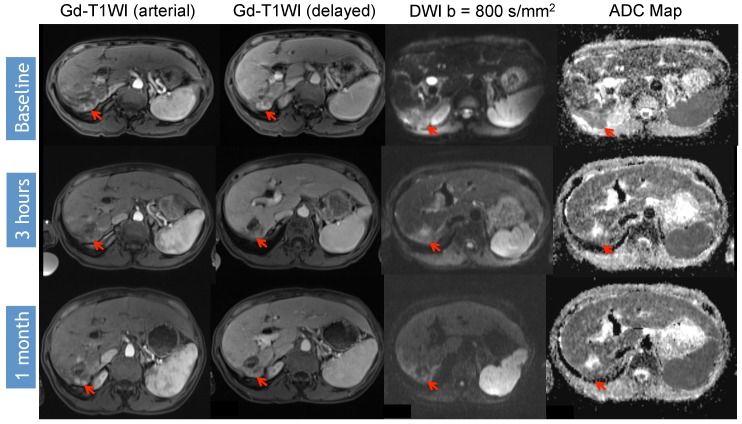
DWI Response Assessment after DEB-TACE treatment. 55-year-old woman presented with a segment VII HCC (arrow) previously treated with DEB-TACE without macrovascular invasion or extrahepatic disease. Note that there is initial absence of enhancement at 3 h, but the area continues to demonstrate restricted diffusion (ADC 0.904 × 10^−3^ mm^2^/s). At one month, the same area now demonstrates enhancement with increased restricted diffusion, findings consistent with progression of disease (PD) (baseline ADC 0.904 × 10^−3^ mm^2^/s, post-3 h ADC 1.02 × 10^−3^ mm^2^/s, post-one month ADC 0.830 × 10^−3^ mm^2^/s).

Identifying early disease progression or absence of response to locoregional therapies is imperative and allows for individualized therapeutic strategies and potentially improved overall prognosis in patients. Volumetric ADC changes after conventional TACE (cTACE) have been investigated at one month after therapy and correlated with six-month RECIST and mRECIST objective responses [27]. Increases in volumetric ADC values to 1.6 × 10^−3^ mm^2^/s in at least >39.8% of the tumor volume correlates with objective response by mRECIST at six months with a sensitivity of 88.4% and specificity of 78.6% (*p* = 0.001). Similar results were obtained by using RECIST response criteria, albeit with lower sensitivity and specificity [27]. An absolute increase in ADC values has also been observed in responding lesions compared to non-responders by mRECIST criteria for cTACE [28]. Kokabi *et al.* [29] demonstrated a % ADC increase for responders *vs.* non-responders (36.4% *vs.* 7.4%; *p* < 0.001) 3 h after DEB-TACE intervention, which further increased after one and three months (98.1% and 115.2%) for responders, whereas no relevant increase for non-responders (−0.1% and 2.1%, *p* > 0.05) was evident. Exemplary diffusion-weighted imaging cases from this study with complete response (Figure 1), partial response (Figure 2), and progressive disease (Figure 3) are illustrated below. A significant percent increase in ADC values has also been reported by another study in responding lesions one and three months post-DEB-TACE compared to non-responders by mRECIST and EASL criteria [30].

In addition, absolute ADC values are capable of differentiating between viable/contrast-enhancing (1.42 ± 0.25 × 10^−3^ mm^2^/s) and necrotic/non enhancing (2.22 ± 0.31 × 10^−3^ mm^2^/s; *p* < 0.001) tumor areas when compared to contrast enhancement patterns 6–8 weeks after cTACE [28]. Yuan *et al.* [31] reported a threshold of 1.84 × 10^−3^ mm^2^/s, which can help for differentiating necrotic *vs.* non-necrotic tumor areas with 92.3% sensitivity and 100% specificity. Average tumor ADC value has also been shown to correlate with the degree of necrosis (*r* = 0.58; *p* < 0.001); a finding that has been corroborated in other studies performed with histopathological specimens [10,11,32,33] (Section 3.4).

#### 3.1.2. Yttrium-90 (Y90)-Radioembolization

Several studies have investigated DWI for response assessment after Y90-radioembolization. Absolute ADC value changes have been shown to be an imaging biomarker for an early response assessment in patients with HCC and portal vein thrombosis (PVT) [34]. Objective mRECIST responders after three months had a significantly greater mean ADC increase after one month (Mean ± standard deviation (SD); from 0.89 (± 0.06) to 1.27 (± 0.14) × 10^−3^ mm^2^/s (% change in ADC: 50.6% ± 20.3%)) than non-responders (from 0.84 (± 0.08) to 1.05 (± 0.13) × 10^−3^ mm^2^/s (% change in ADC: 20.3% ± 5.5%); *p* = 0.001). Overall, the mean % change in ADC increase after one month in responders was significantly higher than in non-responders (*p* = 0.002). % ADC increase three months post Y90 was also significantly higher in responders (46.4%) compared to non-responders (18.4%; *p* = 0.007). In addition, an increase of >30% in the ADC value after three months did predict object treatment response with 90% sensitivity and 100% specificity when compared to the reference response criterion mRECIST [34]. Additional data suggest that DWI after 30 days correlates with the size-based WHO response criteria after 90 days, with sensitivity and specificity rates of 93% and 100% respectively. As a modification to the WHO criteria however, the authors considered a tumor size reduction of only >5% as treatment response [35]. Other case series have demonstrated that a % ADC standard deviation change significantly correlated with tumor response after one and three months [36].

No statistically significant difference between Gd-MRI, DWI, and the combination of both in terms of diagnostic accuracy was evident. In some cases, however, DWI provided additional information to detect HCC progression, which was missed by Gd-MRI alone [37].

There are however conflicting reports about the use of DWI as an imaging response evaluation tool. One case series did not find a statistical significant difference between measured mean ADC of responders and non-responders three months after intervention when compared to EASL criteria [38]. Another cohort of patients that underwent therapy with radioembolization ± Sorafenib in a randomized study did not demonstrate any differences in the median ADC value [17]. In addition, the classification between responders (>5% ADC increase) and non-responders did not predict pathological results. The authors however suggested that, despite the lack of sufficiently differentiating between responders and non-responders, DWI evaluation of tumors with irregular enhancing patterns may be helpful in improving classification of tumor response by increasing sensitivity, specificity and positive/negative predictive values [17].

#### 3.1.3. Local Ablative Therapies

The value of DWI for tumor response assessment after tumor ablation is usually limited by the commonly occurring post treatment hemorrhage, which is known to potentially mask the post-treatment expected ADC increase [39,40]. DWI has been studied in irreversible electroporation (IRE) in a recent case series that showed that ADC values do not demonstrate a relevant change shortly after therapy [12]. Later time points at six and 12 months have not been investigated, although histopathological necrosis has been described to be present after a mean of 10 months (range: 3–17 months) [41]. Therefore, the described histopathological changes after IRE may be detectable by an increase in ADC values at later time points after treatment. In contrast to IRE, another report demonstrated an overall significant ADC value increase 1 and 6 months after radiofrequency ablation (RFA) but not after three months [13].

### 3.2. DWI for Prediction of Overall Survival (OS), Progression Free Survival (PFS) and Recurrence Free Survival (RFS)

Several authors have investigated the value of DWI for overall survival (OS) prediction. A recent study [29] determined that a three-hour post-DEB-TACE ADC increase of ≥20% is associated with significantly prolonged OS (25.4 *vs.* 13.3 months; *p* = 0.017). Bonekamp *et al.* [42] found that the 25th percentile survival for objective responders (≥25% ADC value increase) was significantly longer than for non-responders (11.1 *vs.* 4.9 months; *p* = 0.01) when measured 3–4 weeks after cTACE/DEB-TACE. This threshold remained significant on multivariate analysis (hazard ratio: 0.27; *p* = 0.02). Additionally, the % ADC changes, reduction of enhancement in Gd-MRI during the portal venous phase of ≥65% after treatment has proven to be a significant predictor of OS (25th percentile OS: 11.5 *vs.* 5.1 months; *p* = 0.012). The combination of both parameters further improved the certainty of survival prediction with a 25th percentile OS of 30 months for dual-responder responder (DWI and Gd-MRI response), six months for single-responder (DWI or Gd-MRI response only), and 5.1 months for non-responder (*p* = 0.01) [18,42]. The combination of both parameters was superior to RECIST, mRECIST, and EASL criteria [18]. Another study found an absolute ADC increase within the first 24 h after treatment of at least 0.2 × 10^−3^ mm^2^/s (~14.7% ADC increase) to be associated with prolonged median OS (32 *vs.* 21 months; *p* = 0.002) [43]. Similarly, one month after radioembolization of infiltrative HCC with portal vein thrombosis, an ADC increase of more than 30% was strongly associated with prolonged OS (13.9 *vs.* 5.5 months; *p* = 0.003) [34]. On the other hand, another study failed to predict survival for responders and non-responders when setting the cut-off at 13.6% increase in ADC value at one month following cTACE/DEB-TACE (*p* = 0.07) [44].

One month after cTACE/DEB-TACE intervention, Vandecaveye *et al.* [44] measured ADC values of up to five lesions and classified an ADC increase of at least 13.6% as treatment response. The five months progression free survival (PFS) rate was significantly higher in patients with an average ADC increase of all measured lesions of at least 13.6% (87.5%) compared to an average ADC increase below 13.6% (0%; *p* < 0.001). Interestingly, the five month PFS rate was also significantly lower when only one of the five measured lesions could be classified as non-responding compared to treatment response of each individual lesion (11.8% *vs.* 92.3%; *p* < 0.001). Comparable results on tumor relapse prediction within 3 months after treatment (76.9% sensitivity; 100% specificity; 91.7% accuracy) were reported using the same threshold of 13.6% ADC increase after 5–7 days post-cTACE treatment [45].

### 3.3. How Early Can DWI Changes be Determined for Response Prediction?

An earlier prediction of response is clinically useful in allowing prompt changes to treatment strategies. Several investigators conducted studies to assess DWI changes occurring within the first month after locoregional therapies and to evaluate its prognostic relevance.

Variable timelines in terms of changes in DWI were reported with some studies demonstrating significant changes in treated lesions within 24 h [29,43,46], 2–3 days [10] or 5–7 days [45] after therapy, all of which could predict the outcome. However, another study only detected a significant ADC change following 1–2 weeks after treatment but not before or after that time period [47]. Kokabi *et al.* [29] demonstrated a continuous increase of obtained ADC values during the course of three months for objective treatment responders but not for non-responders after DEB-TACE (Figure 1, Figure 2 and Figure 3). Chung *et al.* [46] investigated intraprocedural DWI changes following cTACE and did not detect a significant % ADC change (3%; SD: ±16.4%) in treated lesions. However, an intraprocedural relative ADC value increase or decrease of ≥15% of individual lesions was associated with a 100% positive predictive value, a specificity of 100%, and a sensitivity of 46% for tumor response after 1 month (EASL) following cTACE.

### 3.4. Correlation of DWI and Tumor Necrosis after Locoregional Therapies

Several studies have compared DWI findings with the degree of histopathological tumor necrosis. Chapiro *et al.* [32] evaluated volumetric ADC changes after cTACE treatment, which showed strong correlation with histopathological findings (*R*^2^ = 0.9662). Others found a significant association between the degree of histopathological necrosis and an increase in absolute ADC values at one month [10] and at three months [11,33] as well as correlation between the degree of necrosis and increase in % ADC at one month [44] post cTACE. In contrast to cTACE, one study failed to reliably predict tumor necrosis extent with DWI after radioembolization [17].

A complete pathologic necrosis (CPN) after treatment is an indisputable prognostic marker associated with beneficial outcomes such as prolonged time to tumor progression (TTP) and OS [44,48,49]. Two studies after cTACE evaluated the value of DWI for detection of CPN. Although one study determined a sensitivity of 75% and specificity of 88% for prediction of complete tumor necrosis after cTACE by measuring the ADC value, no significant difference between partial and complete necrosis could be determined (1.78 × 10^−3^ mm^2^/s; SD: ±0.36 *vs.* 2.32 × 10^−3^ mm^2^/s; SD: ±0.48; *p* = 0.06) [33]. One month post-cTACE, the volumetric ADC measurement was not able to identify any of the CPN lesions as such correctly. Nonetheless, histopathological CPN lesions were classified as at least 95% necrotic by ADC measurement in 86% of cases and as at least 99% necrotic in only 14% of cases [32]. Comparable results were obtained after radioembolization where the mean ADC values were not capable of discriminating between complete and partial tumor necrosis [17,36]. Furthermore, the measured ADC value standard deviation could not significantly differ between lesions with partial (SD: ±63.8) and complete tumor necrosis (SD: ±13.7); *p* = 0.67 [36]. Even the combined evaluation of mRECIST and ADC measurement, could not reliably identify CPN lesions [36]. Despite the non-significant findings results for histopathological correlation of DWI after radioembolization, it has to be noted that none of the studies that successfully could predict the treatment outcomes with DWI measurements have correlated their results with histopathological specimens [34,35].

### 3.5. Pretreatment DWI Assessment and Tumor Response Prediction

A reliable response prediction prior to treatment with locoregional therapies can help to identify suitable patients. The patients without potential treatment benefit, as a result, can directly undergo a more appropriate treatment. Thus, non-beneficial treatments, delayed until appropriate treatment, and the associated healthcare costs, can potentially be reduced. Several studies have investigated diffusion-weighted imaging (*i.e.*, ADC values) as predictor of tumor response of locoregional therapies with conflicting results (Table 1).

Overall, reasons for opposing results and biological mechanisms explaining the rationale behind diffusion parameters and response prediction may only be speculated. It is well acknowledged that the HCC response to intra-arterial therapies heavily relies on the effective drug delivery via the tumor vessels. Thus, drug delivery and tumor response positively correlates with an increased vascularization degree on cross-sectional imaging [50,51]. Furthermore, it has been postulated that lesions with a higher vascularization degree exhibit a more restricted perfusion (*i.e.*, lower ADC values) [52]. This could explain the better outcomes in some studies, which have shown lower pretreatment ADC values to be associated with a better outcome [29,30,43,53].

On the other hand, micro-capillary tumor perfusion may also increase the determined ADC value when lower *b*-values are used, resulting in “false” high readings [52]. Lesions with higher ADC values may also represent partly necrotic lesions, which can be interpreted as a sign of tumor aggressiveness [11,29,43,53]. By nature, necrotic regions are poorly perfused areas resulting in not only a reduced drug delivery dosage than normal but also in an attenuated anti-tumoral effectiveness of chemotherapeutic agents due to the hypoxic/acidic environment. As a result, elimination of tumor cells in this region is less effective [54] and, therefore, could explain the inferior outcomes in lesions with higher pretreatment ADC values.

Compared to intra-arterial therapies, local ablation methods do not rely on vascularization, and are usually limited by tumor size. A recent study from Mori *et al.* [55] investigated pretreatment ADC values in small (≤3 cm) and hypervascular HCC lesions treated with RFA, and found that lower ADC values are an independent predictor for increased tumor recurrence and shorter overall survival rates.

Overall, the comparison between studies and their findings is limited due to different MRI techniques, treatment modalities, and many other factors. Noticeably, the studies reporting on intra-arterial treatment, which could not demonstrate pretreatment ADC value to predict positive outcomes [28,45], investigated relatively smaller tumors (2.0 and 3.14 cm) than other studies (Table 1).

### 3.6. DWI and Tumor Recurrence Detection after Locoregional Therapies

After treatment with locoregional modalities such as cTACE, the incidence of local tumor recurrence is still relatively high. Thus, early peritumoral recurrence detection is a crucial component of the follow-up protocol. For recurrent HCC lesions, the arterial hypervascularity in the vicinity of the treated HCC lesion can be the only distinguishable imaging feature from the background liver tissue in contrast-enhanced cross sectional imaging. However, differentiation between tumor recurrence and benign post-procedural changes can be challenging.

In direct comparison with gadolinium-enhanced MRI, diffusion-weighted imaging has demonstrated a significant lower sensitivity (60.7% *vs.* 82%; *p* < 0.05) and comparable high specificity for tumor recurrence detection. The overall detection accuracy (area under the curve (AUC)) was also significantly lower for DWI than for gadolinium-enhanced MRI (0.74 *vs.* 0.92; *p* < 0.05) [56]. On the other hand, DWI can improve recurrence detection accuracy when evaluated in addition to gadopentetate dimeglumine-enhanced multiphasic dynamic MR images in individual cases [57]. In general, however, the combination DWI and Gd-MRI increased the sensitivity compared to Gd-MRI alone (92% *vs.* 85%; *p* = 0.125) only to a non-significant degree while specificity decreased from 65% to 50%. Therefore, DWI is only of little to no value for recurrence detection according to the current literature.

### 3.7. Limitations of DWI for Locoregional Therapies

Despite of the promising potential of diffusion-weighted imaging to predict and to evaluate tumor response for locoregional therapies, DWI has still some limitations that need to be addressed. Reliable reproducibility and low variances of acquired DWI measurements is key to enable meaningful comparability of HCC lesions between pre-and post-treatments, between different patients, and between institutions. Despite this knowledge, diffusion-weighted imaging reproducibility of HCC lesions has not been adequately evaluated.

One study specifically tested short-term reproducibility of the ADC values in hepatic lesions. When 16 *b*-values were used for ADC measurement, no significant difference between measurements was evident (*p* = 0.65), thus demonstrating a fair reproducibility. However, ADC values differed significantly between measurements when only four *b*-values were used (*p* = 0.01). Nonetheless, regardless of the number of *b*-values measured, ADC value measurement error was still estimated to be between 12%–16% [58]. Another study on malignant hepatic lesions (~90% HCC lesions) even reported a measurement error of up to ~30% [59]. Therefore, determined % ADC changes between pre-treatment and post-treatment measurements might not truly reflect treatment-associated alterations when the % ADC change is below the possible measurement error (12%–30%). Most of the reported cut-offs and thresholds determined by reported studies, to distinguish e.g., between responders and non-responders, however, lie within these reported ranges. This knowledge might explain the considerable overlap of ADC values between study subgroups and why some studies failed to prove statistical difference.

**Table 1:** Overview of studies with pretreatment ADC value assessment of HCC lesions for response and outcome prediction.

Author	Treatment Modality	Number of Patients/Lesions	Lesion Size (in cm)	*b*-Values (s/mm^2^)	ADC Value Responders (× 10^−3^ mm^2^/s)	ADC Value Non-Responders (× 10^−3^ mm^2^/s)	*p*-Value	Tumor Response/Outcome Variable after Treatment	Conclusion
Kokabi 2015 [30]	DEB-TACE	57 patients, 62 tumors	5.8 (SD: ±3.4)	50, 400, 800	0.731 (SD: ±0.201)	1.057 (SD: ±0.215)	*p* = 0.031	mRECIST, EASL, survival	Pretreatment ADC value below the threshold (0.83 × 10^−3^ mm^2^/s) predicts tumor response; Sensitivity (91%) and Specificity (96%).
Kokabi *et al.* 2015 [29]	DEB-TACE	12 patients	<3 (25%), 3–7 (58%), >7 (17%).	50, 400, 800	0.73 (SD: ±0.2)	1.06 (SD: ±0.21)	*p* < 0.001	EASL, mRECIST	Lower baseline ADC values may predict objective tumor response.
Mannelli *et al.* 2013 [11]	cTACE	36 patients, 47 tumors	4.4 (range: 1.0–14.1)	0, 50, 500	1.64 (SD: ±0.39)	1.35 (SD: ±0.42)	*p* = 0.042	>/<50% tumor necrosis on late arterial phase (MRI)	Pretreatment ADC value above the threshold (1.24 × 10^−3^ mm^2^/s) predicts tumor response; Sensitivity (82.3%; 95% CI: 65.5%–93.2%) and Specificity (53.8%; 95% CI: 25.1%–80.8%).
Dong *et al.* 2012 [43]	cTACE	23 patients	7.0 (SD: ±1.7)	0, 500	N/A *	N/A *	-	Median survival	Pretreatment ADC value below the threshold (1.3 × 10^−3^ mm^2^/s) predicts median survival: 31 months (range: 12–36) *vs.* 23 months (range: 9–32); *p* = 0.007.
Yuan *et al.* (2010) [53]	cTACE	27 patients, 34 tumors	7.3 (SD: ±3.3)	0, 500	1.294 (SD: ±0.185)	1.726 (SD: ±0.323)	*p* ≤ 0.001	MRI and CT follow-up and hepatic function **	Pretreatment ADC value below the threshold (1.618 × 10^−3^ mm^2^/s) predicts tumor response; Sensitivity (96%) and Specificity (77.8%).
Kubota *et al.* (2010) [45]	cTACE	25 patients, 36 tumors	2.0 (range: 0.8–5.2)	0, 500	1.222 (SD: ±0.355)	1.357 (SD: ±0.46)	*p* = 0.33	Tumor relapse	Pretreatment ADC value does not significantly predict early tumor relapse.
Sahin *et al.* 2012 [28]	cTACE	22 patients, 77 tumors	3.14 (range: 1.0–15.5)	50, 400, 800	N/A	N/A	-	mRECIST	Pretreatment ADC value does not predict tumor response (*p* = 0.81).
Mori *et al.* 2015 [55]	RFA	136 patients, 168 tumors	2 (SD: ±0.6)	50, 800	0.88 (SD: ±0.46)	0.76 (SD: ±0.28)	0.047	Tumor recurrence, survival	Hypointense ADC map lesions compared to liver parenchyma have a lower cumulative (50% *vs.* 79% ; *p* < 0.001) and local (7% *vs.* 18%; *p* = 0.014) recurrence rate 2 years after treatment than non-hypointense lesions. The 3-year survival rate was significantly longer in non-hypointense (82%) than hypointense (60%) lesions (*p* = 0.007).

Overview of studies evaluating pretreatment ADC values as a predictive factor for tumor response. Tumor response is defined as overall response (complete + partial tumor response) according to stated criteria if not stated otherwise (e.g., survival, tumor relapse). ADC (Apparent Diffusion Coefficient), cTACE (conventional Transarterial Chemoembolization), DEB-TACE (Drug-Eluting Bead Transarterial Chemoembolization), EASL (European Association for the Study of the Liver guidelines), mRECIST (modified Response Evaluation Criteria in Solid Tumors), N/A (Not Available), SD (Standard Deviation), RFA (Radiofrequency Ablation). * Dong *et al.* (2012) [43]: Pretreatment mean ADC value of all lesions was 1.36 × 10^−3^ mm^2^/s; ** Yuan *et al.* [53] response criteria: “follow-up MR and CT imaging and measurement of hepatic function and tumor markers (*i.e.*, >50% decrease in the product of the longest diameter and length of the perpendicular diameter of the lesion or >50% increased necrosis of previous lesions as assessed with contrast agent-enhanced CT or MR imaging)”.

Furthermore, DWI is susceptible to a number of artifacts that can negatively affect image quality and hinder meaningful interpretation. Several studies have reported that lesions located in the left hepatic lobe are more susceptible to ghosting and blurring of images arising from cardiac pulsation compared to the right liver lobe, making ADC measurements unreliable [22,60]. Although electrocardiographic triggering of image acquisition has the potential to reduce cardiac motion artifacts, it is time-consuming and not commonly employed [58,61].

Breath holding for several seconds may not be feasible under certain physical conditions. Therefore, free breathing techniques can be applied with comparable success or even increased reproducibility and improved image quality [40,52,62,63]. Moreover, artifacts due to magnetic field inhomogeneities, especially in fast imaging techniques like echo-planar imaging, and artifacts caused by air-tissue (lung-liver) interferences or fat-water interfaces can also significantly attenuate the validity of the obtained images [40].

**Figure 4:**
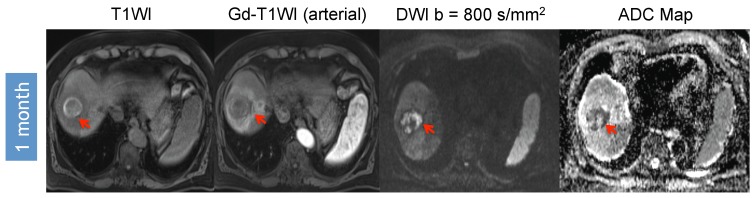
Hemorrhage causes “pseudo-restriction” on DWI. 62-year-old man with segment VIII HCC (arrow) without macrovascular invasion or extra-hepatic disease who underwent radioembolization segmentectomy therapy. There is no Gd-enhancement on one month post-procedural MRI. However, there is significant hyperintensity on pre-contrast images. Although the absence of enhancement correlates with absent diffusion restriction, the presence of hemorrhage creates “pseudo-restriction” without the presence of viable tumor. This pitfall has to be recognized.

Furthermore, more technically-oriented challenges, biologic properties and alterations of the tumor can affect the diffusion estimate of the ADC method. As an example, post-treatment hemorrhage of the tumor may mask the treatment related ADC value increase caused by tumor necrosis, but can be detected with conventional MRI as illustrated in Figure 4 [39,40]. Depending on the settings, the ADC values can be substantially “contaminated” by capillary microperfusion when lower *b*-values and a mono-exponential model, which does not represent “pure diffusion”, are used. The microperfusion fraction can be reduced but not eliminated by using higher *b*-values ≥50 s/mm^2^ for the ADC calculation. Since the perfusion fraction of the HCC lesions cannot be predicted and may vary between tumors and different time points, it can represent a source for variations of measured ADC values [52,60].

None of the presented studies has investigated the cost/benefit ratio of DWI for the response prediction and/or evaluation of locoregional therapies. However, DWI usually comes at the expense of a prolonged scanning time than regular Gd-enhanced MRI or contrast enhanced computed tomography, especially when several ADC values are measured which increases the imaging costs [23]. Furthermore, DWI is usually acquired in addition to contrast enhanced cross sectional imaging techniques. A cost/benefit analysis is therefore warranted for further evaluation of DWI in the clinical setting. Moreover, DWI is currently mainly used in many specialized centers representing an integral part of liver disease diagnosis and treatment follow-up assessment. However, DWI is still not broadly available yet in clinical routine limiting adoption of DWI imaging for locoregional therapy assessment [22,23].

## 4. Conclusions

Diffusion-weighted imaging represents a promising non-invasive diagnostic tool for the evaluation of HCC treatment responses to locoregional therapies. ADC value changes have been shown to occur early after treatment and correlate well with tumor necrosis. These changes are usually evident before changes in tumor size and enhancement, which can provide helpful information for clinical management of patients. Pretreatment ADC values may also have the potential to predict tumor response to locoregional therapies. Nonetheless, it should be noted that DWI can be difficult to interpret since this technique is prone to several artifacts. Thus, diffusion-weighted imaging should be used in conjunction with conventional MRI techniques and diagnostic imaging modalities to reduce misinterpretation.

Despite the promising experimental results, data on DWI for response assessment can still be highly heterogeneous, most likely due to differing study protocols and MR hard- and software used. This hampers direct comparison between studies and limits quick universal adoption. Prospective multicenter studies are required to further evaluate and validate DWI for its diagnostic accuracy, ideal measurement time, and additional technical refinements.
